# Isolated and Community Contexts Produce Distinct Responses by Host Plants to the Presence of Ant-Aphid Interaction: Plant Productivity and Seed Viability

**DOI:** 10.1371/journal.pone.0170915

**Published:** 2017-01-31

**Authors:** Ernesto Oliveira Canedo-Júnior, Graziele Silva Santiago, Luana Fonseca Zurlo, Carla Rodrigues Ribas, Rafaela Pereira Carvalho, Guilherme Pereira Alves, Mariana Comanucci Silva Carvalho, Brígida Souza

**Affiliations:** 1 Laboratório de Ecologia de Formigas, Programa de Pós-Graduação em Ecologia Aplicada, Setor de Ecologia e Conservação, Departamento de Biologia, Universidade Federal de Lavras, Lavras, MG, Brazil; 2 Programa de Pós-Graduação em Entomologia, Departamento de Entomologia, Universidade Federal de Lavras, Lavras, MG, Brazil; Helmholtz Zentrum Munchen Deutsches Forschungszentrum fur Umwelt und Gesundheit, GERMANY

## Abstract

Ant-aphid interactions may affect host plants in several ways, however, most studies measure only the amount of fruit and seed produced, and do not test seed viability. Therefore, the aim of this study was to assess the effects of the presence of ant-aphid interactions upon host plant productivity and seed viability in two different contexts: isolated and within an arthropod community. For this purpose we tested the hypothesis that in both isolated and community contexts, the presence of an ant-aphid interaction will have a positive effect on fruit and seed production, seed biomass and rate of seed germination, and a negative effect on abnormal seedling rates, in comparison to plants without ants. We performed a field mesocosm experiment containing five treatments: Ant-aphid, Aphid, Community, Ant-free community and Control. We counted fruits and seeds produced by each treatment, and conducted experiments for seed biomass and germinability. We found that in the community context the presence of an ant-aphid interaction negatively affected fruit and seed production. We think this may be because aphid attendance by tending-ants promotes aphid damage to the host plant, but without an affect on seed weight and viability. On the other hand, when isolated, the presence of an ant-aphid interaction positively affected fruit and seed production. These positive effects are related to the cleaning services offered to aphids by tending-ants, which prevent the development of saprophytic fungi on the surface of leaves, which would cause a decrease in photosynthetic rates. Our study is important because we evaluated some parameters of plant fitness that have not been addressed very well by other studies involving the effects of ant-aphid interactions mainly on plants with short life cycles. Lastly, our context dependent approach sheds new light on how ecological interactions can vary among different methods of crop management.

## Introduction

Plants are relatively immobile organisms and so they are intimately linked to the physical conditions of their environment and how it varies over the time [1]. However, the biotic community present in a given environment can also deeply affect a plant community. Several organisms, from microscopic [2] to large herbivores [3], can affect, both directly and/or indirectly, plant occurrence, evenness and productivity. Such organisms may affect plants indirectly by causing changes to environmental conditions [4, 5], or directly through disease, parasitism, competition and herbivory [6]. Among the many organisms that can affect plants, insects are of particular importance mainly because of their impact on the yield of cultivated plants, and so they are frequently considered major pests in those systems [7,8].

Ants are present in almost all environments [9] and may occur in basically all kinds of crops, independent of their isolation from natural areas [10]. In fact, ants stand out as one of the most abundant arthropods in crop environments [11]. Ants play a key role in several ecological processes [12], such as the incorporation of organic matter into the soil [13, 14], predation on other arthropods [15] seed dispersal [16, 17] and as a mutualistic partner of plants [18], some caterpillar families [19] and honeydew producing insects [20, 21].

Ants may affect plants directly, such as herbivory from fungus-growing ants [13] or protection against herbivores by plant-tending ants [22, 23], or indirectly, such as protection of mutualistic hemipterans [20, 24, 25] or decreasing visits of pollinators [26].

Among ant interactions that occur on plants, the ant-aphid interaction is probably one of most dynamic since it can vary from being mutualistic to being antagonistic [27, 28]. Ants may benefit from tending-aphids, with the benefits increasing with increasing obligation to the interaction. Ants protect tending-aphids from predators and parasitoids, while they collect honeydew from aphid bodies and plant leaves. Together, these services that the ants provide ensure the aphids of a higher reproductive rate and greater longevity. However, ant attendance may affect aphids negatively since, in some cases, aphids may need to increase their rate of food consumption and change the composition of the honeydew to become more attractive to ants [29], and energy invested in honeydew quality may cause a decrease in aphid development and reproduction [30, 31]. The presence of ants may also decrease the mobility of wingless aphids [32, 33] and the production of winged dispersal forms. Furthermore, ants can prey on tending-aphids, mainly in populations with high density [28] or in the presence of an alternative carbohydrate source [34].

The presence of aphid-tending ants may deeply alter the food web, affecting several trophic levels including the host plant [35]. The presence of tending-ants may decrease the abundance of other herbivorous insects on the host plant [36], as well as decrease aphid predator abundance [37] and decrease [38] or increase [39] aphid parasitoid abundance. However, the effects of ants, mediated by aphids, on host plants are poorly understood, especially with regard to fruit and seed production and seed viability of plants with short life cycles.

Therefore, studies that help elucidate whether the presence of an ant-aphid interaction is detrimental or not to plant productivity and seed viability are urgently needed, since seed viability has been neglected in most previous such field studies. This knowledge will almost certainly be helpful for improving biological control programs especially since the presence of ant-aphid interactions in crops is considered to be one of the major challenges to the success of aphid biological control [40].

In this context the aim of this study was to assess the effects of ant-aphid interaction upon host plant productivity and seed viability in two different conditions: isolated and community contexts. We tested the hypothesis that in both isolated and community contexts, the presence of an ant-aphid interaction will have a positive effect on fruit and seed production, seed biomass and rate of seed germination, and a negative effect on rate of abnormal seedlings, when compared to plants without the interaction (no ants).

We showed that the effects of the presence of an ant-aphid interaction on host plant productivity depend on the ecological context, which can lead to opposite results. Seed viability, the final outcome of plant fitness, was not affected by ant-aphid presence, indicating that the effects of this interaction are not obvious, as previously reported. To our knowledge, this is the first effort to evaluate plant fitness (seed viability) in the context of ant-aphid interaction, and its dependence on ecological context, thus shedding new light on how ecological interactions affect host plants.

## Material and Methods

### Experimental design

We conducted a mesocosm experiment in an organic management area of the sector of Olericulture of Universidade Federal de Lavras (UFLA), Minas Gerais, Brazil, from July to October of 2013. The host plant used for the experiment was the common bean (*Phaseolus vulgaris*). The common bean is an annual herb with a short life cycle (from 60 to 120 days). The variety of common bean used in our experiments was the Carioca bean (“Feijão Carioquinha”), because it is the most cultivated bean variety in Brazil, and has great economic importance in agricultural production in the country [41].

The experimental design included plastic pots containing one bean plantlet each organized into blocks containing five pots, with each treatment being applied to five blocks. Our experiment had five treatments: Control—caged plants without ant-aphid interaction, and exclusion of all arthropods from the plant; Aphid–caged plants infested by aphids, and exclusion of other arthropods, including ants; Ant-aphid–caged plants with presence of the ant-aphid interaction, and exclusion of other arthropods; Community–cageless plants with free access to the entire arthropod community; and Ant free community–cageless plants with ant exclusion.

All treatments, except the Control, were infested with two wingless female individuals of *Aphis craccivora*, Koch, 1851 (Hemiptera: Aphididae). This myrmecophilous aphid species is a generalist pest that is widely distributed around the world; they mainly colonize plants of the family Fabaceae, however, they have been reported to attack plants from about 19 different families [42].

Aphid populations were monitored throughout the duration of the experiment and if an aphid population of a plant became extinguished we replaced it with a similar amount of aphids from the other plants. All aphids used in the experiment were experimentally bred and developed on common bean (*Phaseolus vulgaris*) plants under greenhouse conditions in the Entomology Department—UFLA.

To control access of wingless insects (especially ants) and other arthropods to the bean plants we placed a physical barrier (Formifuu®) on the edge of each pot. We also put circles of Voile fabric on the bottom of each pot to prevent insects from digging access tunnels to the plants. To control winged insects we used a protective cage, which prohibited aerial access to plants.

In treatments with caged plants, we buried the rods of the cages so that the bottom edge of the cage was one centimeter from the soil surface, therefore simultaneously allowing ant access while making access difficult for other arthropods. The cages were made of two plastic rings (1m diameter) and three iron rods (1m tall) covered by Voile fabric. To improve the exclusion of ants from treatments with arthropod exclusion, we removed dead leaves, branches and anything that could be used as bridges by ants or other arthropods to access plants. During the experiment all treatments with arthropod exclusion were monitored and any arthropods found were removed by hand.

### Ant sampling

Weekly sampling of ants began one week after the introduction of aphids to the bean plants and continued for ten weeks in the treatments with ant presence (Ant-aphid and Community treatments). The sampling period (ten weeks) represents the time taken until ant-aphid interactions are observed in bean plants. We sampled aphid-tending ants from 8:00 to 12:00, by examining each plant for five minutes and sampling all ants observed tending aphids. In order to avoid sampling bias we alternated the block and plant with which we started each sampling.

### Plant productivity and seed viability

After beans matured, we collected all of them from each plant and placed them in labeled paper bags. We counted the fruits and then opened them to count the seeds. Seeds were taken to Laboratório Central de Sementes–UFLA, where we measured seed biomass and conducted seed germination experiments. To assess seed biomass, we randomly sampled four seeds in each block of each treatment for a total of 20 seeds per treatment. To measure dry weight, seeds were placed in an oven at 100°C for 24 hours, prior to being weighed on a precision scale.

For seed germination experiments, we randomly selected 40 seeds from each block and wrapped groups of ten seeds (subsamples) in filter paper moistened with distilled water and placed them in a germinator with a constant temperature of 25°C for ten days. Assessments were made on the fourth and the tenth days by counting the number of germinated seeds and abnormal seedlings following seed analysis criteria [43]. For seed biomass and the experiments described above, we excluded Ant-aphid and Aphid treatments, since they did not have enough seeds for the experiments.

### Data analysis

To compare the number of fruits and seeds among treatments we used generalized linear mixed effects models (GLMMs), with plants as the random variable and the Poisson distribution for the fruit and seed data set, using lme4 and phia packages of R software [44, 45]. Due to the low number of seeds produced in all treatments there were not enough seeds to evaluate seed biomass per plant, therefore we used generalized linear models (GLMs) using mean seed biomass per block (Gaussian distribution) as the response variable and treatments as the explanatory variable.

For analysis of germination rates we used generalized linear mixed effects models (GLMMs) to compare the number of germinated seeds between treatments with plants as the random variable. To compare the number of abnormal seedlings we constructed generalized linear mixed models (GLMMs), where samples (plants) were considered as subsamples nested in blocks. All analyses were performed in R 2.15.1 software [46], using lme4 and phia packages [44, 45].

## Results

In total we sampled 15 aphid-tending ant morphospecies from nine genera. The most diverse genera were *Camponotus* and *Pheidole*, with three aphid-tending ant morphspecies each, and *Linepithema* and *Brachymyrmex*, with two aphid-tending ant morphospecies each. The genera *Hylomyrma*, *Dorymyrmex*, *Gnamptogenys*, *Ectatomma* and *Crematogaster* had only one ant morphospecies each (S1 Table).

**Fig 1:**
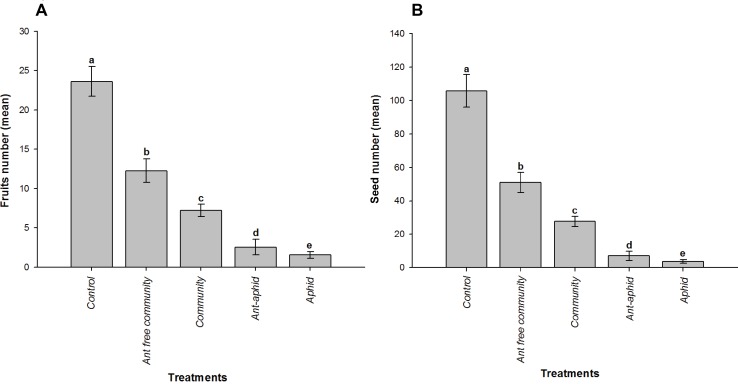
Plants production (fruits and seeds) per treatment. Produced fruits (A) and seeds (B) for the treatments: Control—caged plants without ant-aphid interaction, and exclusion of all the arthropods from the plant; Aphid–caged plants infested by aphids, and exclusion of others arthropods, including ants; Ant-aphid–caged plants with the presence of the ant-aphid interaction only, and exclusion of other arthropods; Communit**y**–cageless plants with free access to the entire arthropod community; and Ant free community–plants with ant exclusion.

We found differences in the number of fruits (X^2^ = 842.24; p< 0.001) and seeds (X^2^ = 4446.3; p<0.001) among treatments, with the control having the greatest production of fruits and seeds. In the presence of the entire arthropod community, the ant-aphid interaction had a negative effect on fruit and seed production in comparison to plants without ants. However, the isolated ant-aphid interaction had a slightly positive effect on fruit and seed production when compared to plants without ants (Fig 1A and 1B).

**Fig 2:**
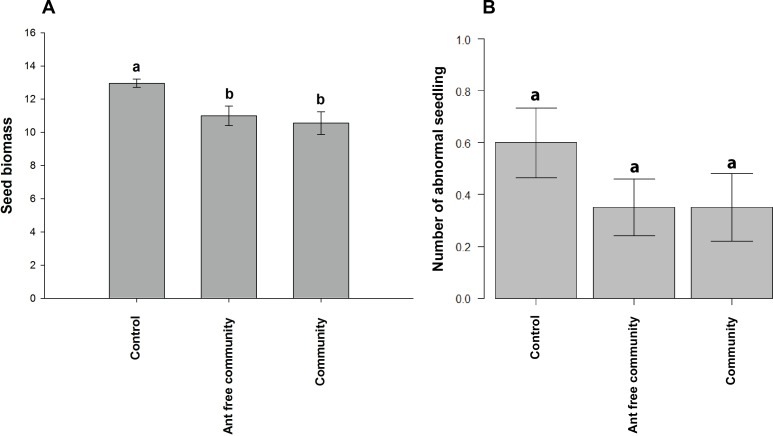
Traits and viability of seeds produced. Seed biomass (A) and number of abnormal seedlings (B) for the treatments: Control—caged plants without ant-aphid interaction, and exclusion of all the arthropods from the plant; Community–cageless plants with free access to the entire arthropod community; and Ant free community–plants with ant exclusion. The isolated treatments Aphid and Ant-aphid did not produce seed amount enough to seed traits experiments.

We did not find enough seeds to carry out seed biomass experiments for the Ant-aphid and Aphid treatments, but for the other treatments we found significant differences (X^2^ = 5.5445; p = 0.01971), with the Control having greater seed biomass than plants in the presence of the entire arthropod community with and without the presence of ants, among which seed biomass was similar (p< 0.05) (Fig 2A).

Almost all seeds germinated irrespective of treatment, so we did not perform an analysis of germination rates (S2 Table). The number of abnormal seedlings was not affected by treatments (X^2^ = 0.1917; p = 0.9086) (Fig 2B).

## Discussion

In our study we assessed the effects of the presence of an ant-aphid interaction on host plant fitness. According to our experiments, the presence of the ant-aphid interaction negatively affected the number of fruits and seeds when in a community context, but did not have an effect on seed biomass and viability. On the other hand, in an isolated context (i.e. without arthropod community), the ant-aphid interaction positively affected fruit and seed production.

We observed that the arthropod community has a strong negative effect on plant fitness, as seen when comparing the control plants (without arthropods and ant-aphid interactions) to all other treatments. These effects can be direct, such as leaf loss caused by chewing insects or sap loss caused by sap-sucking insects, or indirect, such as disease that is transmitted by herbivorous arthropods [47]. Such damage can decrease the availability of energy to be invested in plant reproduction [48, 49, 50].

In other studies that have evaluated the effects of ant-aphid interactions on plant fitness in a community context, the presence of aphid-tending ants had positive effects on host plants. These effects were mediated by aphid protection, since ants can exclude arthropods, including herbivores, which would forage on the plant. Styrsky and Eubanks [51] reported that the presence of aphid-tending ants caused an increase in the production of cotton buds, flowers, seeds and seed mass and Zhang et al. [49] showed that the presence of ants decreased leaf loss by chewing insects.

However, in our experiments the presence of aphid-tending ants caused negative effects on plant fitness when evaluated in a community context, corroborating with Wu et al. [52] and Levan and Holway [26], who found negative effects on plant yield (e.g. number of fruits, seeds, seed mass and seed malformation), and Renault et al. [53], who found a lower number of viable seeds when aphid-tending ants were present. We believe that our results may be related to two factors: the presence of other herbivorous arthropods and the ant-aphid interaction *per se*. Some studies have shown that hemipteran-tending ants indirectly protect host plants from other herbivorous arthropods [54], although this behavior is correlated with ant aggressiveness, which may vary among different ant species and the nutritional requirements of the ant nest [55, 24].

Altfeld and Stiling[56] also pointed out that ants have stronger effects on insects that are directly related to the ant-aphid interaction (e.g. aphid predators, parasitoids and other sap sucking insects), and do not affect all herbivorous arthropods on the host plant. In our experiment we did not control which ant species tended the aphids of the bean plants, which changed over the course of the experiments (Canedo-Júnior in preparation). Thus, different tending-ant species (total of 15 ant species) with their varying intensities of protective behavior, may not all offer efficient protection to a host plant.

The presence of ants could increase the damage caused by aphids to the host plant by increasing aphid density and longevity, and consequently increasing host plant sap loss [53], beyond damages caused by the transmission of the numerous viruses for which aphids can be vectors [57,58]. Therefore, the damages of the ant-aphid interaction, in addition to the damage caused by other herbivorous arthropods, decreases the amount of energy that would be invested in reproduction, which is reflected in fruit and seed production.

Besides the production of fewer fruits and seeds, plants exposed to the arthropod community, irrespective of the presence of an ant-aphid interaction, produced slightly lighter seeds than control plants. Plants may have several strategies for responding to damages caused by herbivory, including both physiological and morphological changes [59, 60], which may directly affect plant reproductive success.

Based in our results, we believe that stressed bean plants produced fewer seeds but invest more energy in seed biomass, since produced seeds had almost the same weight as seeds from the control plants. This result may be related to the fact that among herbaceous plants there may be a correlation between seed size/mass and germinability [61, 62], whereas germination and abnormal seedling rates were not affected by the presence of other arthropods independently of the presence of ants.

For isolated plants (those without other arthropods), aphids had strong affects independently of the presence of ants, since plants with only aphids and with the ant-aphid interaction produced 9 and 15 fold less fruits and 15 and 29 fold less seeds, respectively, than control plants. Without predators, parasitoids and competing herbivores, aphids have energy to invest in reproduction, causing almost exponential increase in aphid population growth [63]. Aphids are known to be a major pest of crops, and especially of herbaceous plants, since they can breed quickly and obtain large population sizes, which cause great damage by draining host plants of resources [64]. Such large populations ingest large amounts of sap with the excess (honeydew) being dropped on host plant leaves, facilitating the development of saprophytic fungi. Development of saprophytic fungi may cover photosynthetic surfaces of leaves, causing losses in primary productivity [65]. In addition to causing losses of plant resources, *A*. *craccivora* can also vector about 30 plants viruses [42].

Even if the presence of ants leads to a larger and more long-lived aphid population, this interaction may benefit host plant. Ants may collect honeydew, both directly from aphids as well as from leaves, and aphid exuviae that may accumulate on leaves, thus cleaning the leaf surface [66]. These cleaning services offered by the ants to the aphids indirectly benefit the host plant, since ant presence decreases the occurrence of saprophytic fungi on plant leaves. Therefore, even plants being damaged by aphids draining resources may not have their photosynthetic rates affected, thereby allowing greater fruit and seed production in comparison to plants with only aphids.

In both experimental contexts (isolated and community) we did not control the ant species on the bean plants, and so we are unable to verify the effect of each specific ant species on the host plant. However, we did show the results for the effects of an entire aphid-tending ant assemblage on host plant fitness, which may be closer to what actually occurs in nature. Although the protection ants offer to tending-aphids may be dependent on ant species, the cleaning services offered probably do not, since the collection of honeydew, independent of being on aphid bodies or on the leaf surface, have the same positive effect on the host plant.

In practical terms, our experiments simulated two distinct methods of organic crop management: the community context of agro-ecological farming, and the isolated context of organic monoculture. Agro-ecological farming has greater biological diversity and smaller management intensity than organic monoculture. Biological diversity lessens the damages to the host plant caused by the ant-aphid interaction due to aphid predation and/or competition with other arthropods [67]. Vegetable diversity may also hamper aphid dispersion and offer more diversity of food sources to ants, decreasing the intensity of ant-aphid interactions [68].

On other hand, in organic monoculture there is great management intensity and lower biological diversity, however, the ant-aphid interaction can occur in these environments independently of management and proximity to natural areas [10]. Therefore, in these environments, due to the lower diversity of arthropods, aphids have fewer enemies and competitors, and thus can cause greater damage and negatively influence reproduction of the host plant independently of the presence of ants, in comparison to the damage to host plants by aphids in presence of an entire associated arthropod community.

To our knowledge, our study is the first to assess the effects of ant-aphid interaction on host plants in two different contexts: isolated and in the presence of an entire associated arthropod community. In an isolated context, the presence of aphid-tending ants has a positive effect on plant productivity, while in a community context ants have a negative effect. Seed viability, a fitness parameter that has been poorly studied in this context, was not affected by the presence of aphid-tending ants. The approach used in our study is important because it shows how the effects of ecological interactions can vary among different methods of crop management, since our results were dependent on ecological context. Evaluating seed viability in response to the presence of ant-aphid interaction is essential for determining whether this interaction has positive or negative effects on host plants, since this parameter is the final outcome of plant fitness.

## Supporting Information

S1 Table Occurrence of aphid-tending ant species in each week sampled. Aphid-tending ants were actively sampled from treatments: Community and Ant-aphid totalizing 50 sampled beans plants (*Phaseolus vulgaris*). A–Occurrence of ant species in Ant-aphid treatment; and B–Occurrence of ant species in Community treatment.
(DOCX)

S2 Table Bean plant seed germination rates. Bean plant seed germination rate, mean per block (ten seed/block), from three treatments: Control—caged plants without ant-aphid interaction, and exclusion of all the arthropods from the plant; Community–cageless plants with free access to the entire arthropod community; and Ant free community–plants with ant exclusion. The isolated treatments Aphid and Ant-aphid did not have produced seed amount enough required to seed traits experiments.
(DOCX)
