# Supplementary material for: Isolated and Community Contexts Produce Distinct Responses by Host Plants to the Presence of Ant-Aphid Interaction: Plant Productivity and Seed Viability
Source: PLoS One. 2017 Jan 31;12(1):e0170915. doi: 10.1371/journal.pone.0170915 (PMC5283668; doi:10.1371/journal.pone.0170915)
Supplement: S1 Table — Aphid-tending ants were actively sampled from treatments: Community and Ant-aphid totalizing 50 sampled beans plants (Phaseolus vulgaris). A–Occurrence of ant species in Ant-aphid treatment; and B–Occurrence of ant species in Community treatment. (DOCX) [file pone.0170915.s001.docx]

**S1 Table. Occurrence of aphid-tending ant species in each week sampled.**

Aphid-tending ants were actively sampled from treatments: Community and Ant-aphid totalizing 50 sampled beans plants (*Phaseolus vulgaris*). A – Occurrence of ant species in Ant-aphid treatment; and B – Occurrence of ant species in Community treatment.

|  | **Week sampled** | | | | | | | | | |
| --- | --- | --- | --- | --- | --- | --- | --- | --- | --- | --- |
| **Ant species** | **1^st^** | **2^nd^** | **3^rd^** | **4^th^** | **5^th^** | **6^th^** | **7^th^** | **8^th^** | **9^th^** | **10^th^** |
| *Linepithema* sp.1 | AC | AC | AC | AC | AC | C | C | AC | AC | AC |
| *Linepithema* sp.2 | AC | AC | AC | AC | AC | AC |  | AC | AC | A |
| *Camponotus* sp.1 |  |  |  |  |  |  |  | A | A | A |
| *Camponotus* sp.3 |  |  |  |  |  | A | AC | A | A | A |
| *Camponotus* sp.4 | C |  |  |  |  | A | A | A | A | A |
| *Hylomyrma* sp.1 | A |  |  |  |  |  |  |  |  |  |
| *Dorymyrmex* sp.1 | AC | AC | A | A | A | A | AC | A | AC | A |
| *Pheidole* sp.1 | C | AC | AC | A | AC | AC | A | C | AC | C |
| *Pheidole* sp.2 | AC | AC | AC | AC | AC | AC | AC | AC | AC | AC |
| *Pheidole* sp.4 | A |  |  |  | A | C | C | A |  | C |
| *Brachymyrmex* sp.1 | AC | A | AC | AC | AC | AC | AC | AC | AC | A |
| *Brachymyrmex* sp.2 |  |  |  | A |  |  |  |  |  |  |
| *Gnamptogenys* sp.1 |  |  |  |  |  | C |  |  |  |  |
| *Ectatomma* sp.1 |  |  |  |  |  | C |  |  |  |  |
| *Crematogaster* sp.1 |  |  |  |  |  |  |  |  | C |  |
