# Supplementary material for: Isolated and Community Contexts Produce Distinct Responses by Host Plants to the Presence of Ant-Aphid Interaction: Plant Productivity and Seed Viability
Source: PLoS One. 2017 Jan 31;12(1):e0170915. doi: 10.1371/journal.pone.0170915 (PMC5283668; doi:10.1371/journal.pone.0170915)
Supplement: S2 Table — Bean plant seed germination rate, mean per block (ten seed/block), from three treatments: Control—caged plants without ant-aphid interaction, and exclusion of all the arthropods from the plant; Community–cageless plants with free access to the entire arthropod community; and Ant free community–plants with ant exclusion. The isolated treatments Aphid and Ant-aphid did not have produced seed amount enough required to seed traits experiments. (DOCX) [file pone.0170915.s002.docx]

**S2 Table. Bean plant seed germination rates**.

Bean plant seed germination rate, mean per block (ten seed/block), from three treatment: Control - caged plants without ant-aphid interaction, and exclusion of all the arthropods from the plant; Community – cageless plants with free access to the entire arthropod community; and Ant free community – plants with ant exclusion. The isolated treatments Aphid and Ant-aphid did not have produced seed amount enough required to seed traits experiments.

| **Treatment** | **Blocks** | **Germination rate (%)** |
| --- | --- | --- |
| Control | 1 | 100 |
| Control | 2 | 100 |
| Control | 3 | 100 |
| Control | 4 | 100 |
| Control | 5 | 100 |
| Ant free community | 1 | 100 |
| Ant free community | 2 | 99.5 |
| Ant free community | 3 | 100 |
| Ant free community | 4 | 100 |
| Ant free community | 5 | 100 |
| Community | 1 | 99.75 |
| Community | 2 | 100 |
| Community | 3 | 100 |
| Community | 4 | 100 |
| Community | 5 | 100 |
